# Genomic Analysis of 15 Human Coronaviruses OC43 (HCoV-OC43s) Circulating in France from 2001 to 2013 Reveals a High Intra-Specific Diversity with New Recombinant Genotypes

**DOI:** 10.3390/v7052358

**Published:** 2015-05-07

**Authors:** Nathalie Kin, Fabien Miszczak, Wei Lin, Meriadeg Ar Gouilh, Astrid Vabret, Epicorem Consortium

**Affiliations:** 1 Normandie Université, 14032 Caen, France; E-Mails: fabienmzz@yahoo.fr (F.M.); meriadeg.le-gouil@pasteur.fr (M.A.G.); a-vabret@chu-caen.fr (A.V.); 2 Université de Caen, Unité de Recherche Risques Microbiens (U2RM), F-14000 Caen, France; E-Mails: wei.lin@hotmail.fr (W.L.); epicorem@pasteur.fr (E.C.); 3 Department of Virology, University Hospital of Caen, F-14033 Caen, France; 4 Institut Pasteur, Environment and Infectious Risks Research and Expertise Unit, F-75015 Paris, France

**Keywords:** genotype, sequencing, coronavirus, phylogeny, recombination, HCoV-OC43

## Abstract

Human coronavirus OC43 (HCoV-OC43) is one of five currently circulating human coronaviruses responsible for respiratory infections. Like all coronaviruses, it is characterized by its genome’s high plasticity. The objectives of the current study were to detect genetically distinct genotypes and eventually recombinant genotypes in samples collected in Lower Normandy between 2001 and 2013. To this end, we sequenced complete nsp12, S, and N genes of 15 molecular isolates of HCoV-OC43 from clinical samples and compared them to available data from the USA, Belgium, and Hong-Kong. A new cluster E was invariably detected from nsp12, S, and N data while the analysis of nsp12 and N genes revealed the existence of new F and G clusters respectively. The association of these different clusters of genes in our specimens led to the description of thirteen genetically distinct genotypes, among which eight recombinant viruses were discovered. Identification of these recombinant viruses, together with temporal analysis and tMRCA estimation, provides important information for understanding the dynamics of the evolution of these epidemic coronaviruses.

## 1. Introduction

Coronaviruses belong to the *Coronaviridae* family in *Nidovirales* order [1]. This order comprises the largest enveloped single-strand RNA viruses, and includes three other families; *Arterividae*, *Roniviridae*, and the most recently described *Mesoniviridae* [2]. Coronaviruses are divided into four genera named *Alpha*-, *Beta*-, *Delta*-, and *Gammacoronavirus*, based on the phylogenetic distance of highly conserved domains [3,4,5]. Alphacoronaviruses are divided into two subgroups or clades, A and B or 1 and 2, and betacoronaviruses are divided into four subgroups or clades, A to D or 1 to 4 [6,7].

Coronaviruses infect a wide range of avian or mammalian species, and are responsible for enteric or respiratory infections [8,9,10]. Six human coronaviruses (HCoVs) have been identified of which four are in circulation: HCoV-229E, HCoV-NL63 (genus *Alphacoronavirus*), and HCoV-OC43 and HCoV-HKU1 (genus *Betacoronavirus* clade A). These four viruses are responsible for mild upper-respiratory tract infections, yet cause more severe respiratory pathologies in infants, immunocompromised patients and elderly people [11,12,13,14,15,16,17,18,19,20,21,22]. The other two human coronaviruses, Severe Acute Respiratory Syndrome coronavirus (SARS-CoV) and Middle-East Respiratory Syndrome coronavirus (MERS-CoV), belonging to *Betacoronavirus* genus, clades B and C respectively, cause severe respiratory pathologies. SARS-CoV circulated in 2002–2003 and caused a global outbreak with more than 8000 cases, and produced a rate of fatal cases of nearly 10%; and MERS-CoV emerged in 2012 in the Middle-East, causing severe pneumonia similar to that from SARS [23,24,25]. To date, 961 laboratory confirmed cases of MERS-CoV infection, including 418 fatal cases, have been reported [26].

The first isolation of HCoV-OC43 from a nasopharyngeal wash was reported in a publication in 1967 [13]. In 2004 and 2005 respectively, St-Jean *et al.* and Vijgen *et al.* published the first complete genome sequence of HCoV-OC43 [27,28]. The genome of HCoV-OC43 consists of a positive sense, single-stranded RNA molecule, which is 30,738 bases in length, encoding 11 ORFs. The first two ORFs (1a and 1b) starting from the 5' end of the molecule account for approximately two-thirds of the genome. The last third towards the 3' end containing genes coding the hemagglutinin esterase (HE), the spike glycoprotein (S), the envelop protein (E), the matrix glycoprotein (M), and the nucleocapsid phosphoprotein (N). The mechanism of replication of HCoV-OC43 uses a low fidelity RNA dependent RNA polymerase (RdRp) characterized by a mutation rate of 10^−3^ to 10^−4^ mutation/nucleotide/round of replication [29,30]. The replication of coronavirus genomes requires a step during which a set of subgenomic RNAs is generated. This mechanism contributes to promoting homologous recombination events [31]. Due to its genomic plasticity, coronaviruses are characterized by a high potential of evolution, adaptation, and interspecies jumping [7]. Coronaviruses are also characterized by their interspecies and intraspecies variability. This study is focused on the latter.

In 2006, Vabret *et al.* observed the co-circulation of genetic variants inside the species HCoV-OC43 by analysing the partial S gene (nt 23,449 to 26,332, reference to HCoV-OC43 ATCC-VR759 AY391777), coding for the glomerular part of the S protein (sub-unit S1) of 7 HCoV-OC43s from clinical samples collected in 2003 at the University Hospital of Caen. They observed four genetically distinct subgroups. One of the subgroups constitutes an outlier group located between HCoV-OC43 and Bovine coronavirus (BCoV), containing a 12-nucleotide deletion in common with BCoV but not with other HCoV-OC43s [32]. More recently, four genetically distinct HCoV-OC43 genotypes have been identified from respiratory specimens sampled at the Public Hospital of China over a 7-year period (2004 to 2011) [33]. In this study, Lau *et al.* based their genotype definition upon the complete sequence of nsp12, S and N genes [33]. Genotype A contains the prototype VR759 strain isolated in 1967. Genotypes B and C are two naturally circulating HCoV-OC43s. Genotype D is a recombinant virus of genotypes B and C, obtained from a specimen from 2004 [33]. Based on a bootscan analysis of the complete genome of the 3 HCoV-OC43s belonging to the circulating genotypes B, C, and D, it was assumed that a hot spot was likely located between the nsp12 and S genes, more precisely at the nsp12/nsp13 junction. Our objective was to investigate the presence, the temporal distribution and the recombination patterns of HCoV-OC43 in Lower Normandy over 13 years (2001 to 2013), using the methodology and the HCoV-OC43 genotype definition elaborated by Lau *et al.* in 2011 [33]. This study focuses on the sequences of the nsp12, S, and N genes of 15 HCoV-OC43s detected in respiratory specimens sampled from 2001 to 2013.

## 2. Results

### 2.1. Sequencing of nsp12, S and N Genes

Of the 15 HCoV-OC43s and the prototype strain VR759, six, eight, and three overlapping sequences were obtained for the nsp12, S, and N genes, respectively. After assembly, these overlapping fragments encompassed the full nsp12, S, and N genes. In this study, all HCoV-OC43s including the VR759 prototype strain are associated with three accession numbers in GenBank, for nsp12, S, and N genes (Table 1). The sequences of HCoV-OC43s were named according to the following nomenclature: Virus/FRA-EPI/location of sampling/year of sampling/specimen number. EPI is an abbreviation for EPICOREM consortium. For the prototype strain, the specimen number is replaced by VR759.

**Table 1:** GenBank accession numbers associated with the sequences used in this study.

Name of Sequences	Accession Number		
	nsp12	S	N
HCoV-OC43/FRA_EPI/Caen/1967/VR759	KF963213	KF963229	KF963245
HCoV-OC43/FRA_EPI/Caen/2001/01	KF963214	KF963230	KF963246
HCoV-OC43/FRA_EPI/Caen/2001/02	KF963215	KF963231	KF963247
HCoV-OC43/FRA_EPI/Caen/2002/03	KF963216	KF963232	KF963248
HCoV-OC43/FRA_EPI/Caen/2002/04	KF963217	KF963233	KF963249
HCoV-OC43/FRA_EPI/Caen/2003/05	KF963218	KF963234	KF963250
HCoV-OC43/FRA_EPI/Caen/2004/06	KF963219	KF963235	KF963251
HCoV-OC43/FRA_EPI/Caen/2005/07	KF963220	KF963236	KF963252
HCoV-OC43/FRA_EPI/Caen/2006/08	KF963221	KF963237	KF963253
HCoV-OC43/FRA_EPI/Caen/2007/09	KF963222	KF963238	KF963254
HCoV-OC43/FRA_EPI/Caen/2008/10	KF963223	KF963239	KF963255
HCoV-OC43/FRA_EPI/Caen/2009/11	KF963224	KF963240	KF963256
HCoV-OC43/FRA_EPI/Caen/2010/12	KF963225	KF963241	KF963257
HCoV-OC43/FRA_EPI/Caen/2011/13	KF963226	KF963242	KF963258
HCoV-OC43/FRA_EPI/Caen/2012/14	KF963227	KF963243	KF963259
HCoV-OC43/FRA_EPI/Caen/2013/15	KF963228	KF963244	KF963260
	**full genome**		
OC43/human/USA/971-5/1997	KF530099		
OC43/human/USA/965-6/1996	KF530098		
OC43/human/USA/832-27/1983	KF530093		
OC43/human/USA/008-5/2000	KF530092		
OC43/human/USA/911-58/1991	KF530091		
OC43/human/USA/931-85/1993	KF530090		
OC43/human/USA/901-54/1990	KF530088		
OC43/human/USA/872-5/1987	KF530086		
OC43/human/USA/951-18/1995	KF530084		
OC43/human/USA/8912-37/1989	KF530073		
OC43/human/USA/925-1/1992	KF530071		
OC43/human/USA/007-11/2000	KF530068		
OC43/human/USA/953-23/1995	KF530062		
HK04_01	JN129834		
HK04_02	JN129835		
19572 Belgium 2004	AY903460		
87309 Belgium 2003	AY903459		
HCoV-OC43 VR759 [28]	NC_005147		
HCoV-OC43 VR759 [34]	AY391777		
BCoV Mebus	U00735		
BCoV Kakewaga	AB354579		
BCoV Quebec	AF220295		

### 2.2. Phylogenetic Analysis of nsp12, S, and N Genes

**Figure 1:**
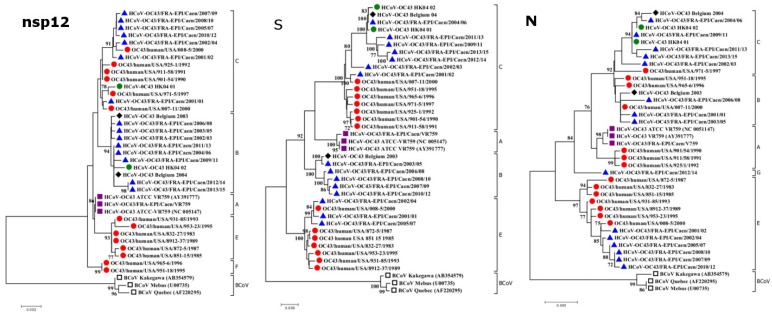
Phylogenetic analysis of the complete nsp12, S, and N genes of the 36 HCoV-OC43 strains and 3 BCoV strains. The phylogenetic trees were constructed by the neighbor joining method [35]. Bootstrap values were calculated from 1000 replicates. Bootstrap values over 70% are shown [40]. The evolutionary distances were computed using the Kimura 2-parameter method (kimura) and units are the number of base substitutions per site. Evolutionary analyses were conducted in MEGA, version 6.06. [41]. Blue triangle, isolates from Caen; red circle, isolates from USA; green circle, isolates from Hong-Kong; black diamond, isolates from Belgium; purple square, ATCC-VR759 strains, empty black square, BCoV strains.

The phylogenetic analysis was conducted by comparing the topology of the three phylogenetic trees obtained from the nsp12, S, and N genes. Figure 1 shows the three trees obtained by the neighbor joining method [35]. On each tree, five to six clusters were observed, including an outlier group compounded with the three BCoVs sequences Mebus, Kakegawa, and Quebec that root HCoV-OC43 sequences [36,37,38]. The clustering is supported with high bootstrap values. We used part of the nomenclature of genotypes proposed by Lau *et al.* in 2011 [33]. These authors described the genotypes A, B, C, and D from nsp12, S and N genes. We used this nomenclature to name each genotype using three letters corresponding to clusters in which the different sequences are located in nsp12, S, and N trees, respectively. Following this nomenclature, genotype D is a recombinant genotype B/C/C. The three previously described clusters—A, B, and C—are observed in our three gene trees in addition to a newly described cluster E. The nsp12 tree depicts a new cluster we named “F” that roots all other HCoV-OC43s. According to the N gene, a sixth cluster we named “G” separates from cluster E and roots clusters A, B, and C. Among the set of sequences obtained from the 16 HCoV-OC43s of our study, three are distributed in two non-recombinant genotypes as follows: the VR759 sequence belongs to the genotype AAA; HCoV-OC43/FRA-EPI/Caen/2003/05 and HCoV-OC43/FRA-EPI/Caen/2006/08 belong to genotype BBB. The 13 other sets of sequences are allocated among six recombinant genotypes as follows: five genotypes BCC, defined as genotype D by Lau *et al.*; two genotypes CEE, three genotypes CBE, one genotype BCG, one genotype CCE, and one genotype CEB. Among the 14 American sets of sequences, seven are distributed into two non-recombinant genotypes as follows: one CCC and six EEE. The seven other sets of sequences are distributed among four recombinant genotypes as follows: three genotypes CCA, two genotypes FCB, one genotype CCB, and one genotype CEE. Among the two sets of sequences from Hong-Kong, one is a non-recombinant genotype CCC and the other is a recombinant genotype BCC. Among the two HCoV-OC43s from Belgium, one is a non-recombinant genotype BBB and the other is a recombinant genotype BCC. Finally, the two last sequences of HCoV-OC43 VR759 (accession number: AY391777 and NC005147) belong to the non-recombinant genotype AAA [28,39]. The results of the phylogenetic analysis are summarized in Table 2.

### 2.3. Insights in the Evolutionary History

**Figure 2:**
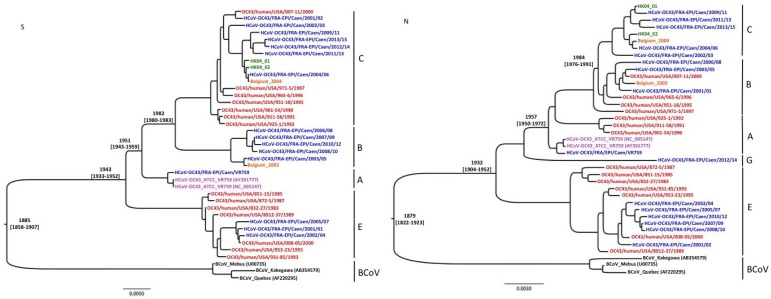
Results of Bayesian analysis based on S and N gene sequence data. Inferences were calculated with the one parametric coalescent model with a constant size, under the TN93+G substitution parameter, using the BEAST package (version 1.8.2) [42]. The length of MCMC was fixed at 10^8^ states for S and N genes. The dates of emergence of mean clusters are indicated, associated with the 95% HPD.

The two alignments constructed from the 39 dated sequences of S and N genes have been used to set up a molecular clock calibration in order to estimate the date of emergence of mean clusters. Figure 2 shows Bayesian trees inferred from the alignments of S and N genes. The dates of emergence of the clusters and the corresponding 95% Highest Posterior Density (95% HPD) are indicated. Based on the sequence data of the S gene, the tMRCA of BCoV and HCoV-OC43 is estimated in 1885, with a 95% HPD from 1858 to 1907. For the HCoV-OC43, cluster E is predicted to have emerged in 1943 (95% HPD 1933 to 1952), and genotype A is estimated to have emerged in 1951 (95% HPD, 1943 to 1959). Genotypes B and C are predicted to have emerged from their tMRCA in 1982 (95% HPD, 1980 to 1983). The molecular clock conducted from sequence data of N gene allows us to estimate the tRMCA of BCoV and HCoV-OC43 in 1879 (95% HPD, 1822 to 1923). Genotypes E and A are predicted to have emerged in 1932 (95% HPD, 1904 to 1952) and 1957 (95% HPD, 1950 to 1972), respectively. Genotypes B and C are predicted to have emerged from their tMRCA in 1984 (95% HPD, 1976 to 1991).

**Table 2:** Results of phylogenetic analysis of complete nsp12, S and N genes. * Genotype D, according to the definition from Lau *et al.* [33].

Sequences	nsp12	S	N	Genotype
**France**				
HCoV-OC43/FRA_EPI/Caen/2001/01	C	E	B	CEB
HCoV-OC43/FRA_EPI/Caen/2001/02	C	C	E	CCE
HCoV-OC43/FRA_EPI/Caen/2002/03	B	C	C	BCC *
HCoV-OC43/FRA_EPI/Caen/2002/04	C	E	E	CEE
HCoV-OC43/FRA_EPI/Caen/2003/05	B	B	B	BBB
HCoV-OC43/FRA_EPI/Caen/2004/06	B	C	C	BCC *
HCoV-OC43/FRA_EPI/Caen/2005/07	C	E	E	CEE
HCoV-OC43/FRA_EPI/Caen/2006/08	B	B	B	BBB
HCoV-OC43/FRA_EPI/Caen/2007/09	C	B	E	CBE
HCoV-OC43/FRA_EPI/Caen/2008/10	C	B	E	CBE
HCoV-OC43/FRA_EPI/Caen/2009/11	B	C	C	BCC *
HCoV-OC43/FRA_EPI/Caen/2010/12	C	B	E	CBE
HCoV-OC43/FRA_EPI/Caen/2011/13	B	C	C	BCC *
HCoV-OC43/FRA_EPI/Caen/2012/14	B	C	G	BCG
HCoV-OC43/FRA_EPI/Caen/2013/15	B	C	C	BCC *
**USA**				
OC43/human/USA/832-27/1983	E	E	E	EEE
OC43/human/USA/851-15/1985	E	E	E	EEE
OC43/human/USA/872-5/1987	E	E	E	EEE
OC43/human/USA/8912-37/1989	E	E	E	EEE
OC43/human/USA/901-54/1990	C	C	A	CCA
OC43/human/USA/911-58/1991	C	C	A	CCA
OC43/human/USA/925-1/1992	C	C	A	CCA
OC43/human/USA/931-85/1993	E	E	E	EEE
OC43/human/USA/953-23/1995	E	E	E	EEE
OC43/human/USA/951-18/1995	F	C	B	FCB
OC43/human/USA/965-6/1996	F	C	B	FCB
OC43/human/USA/971-5/1997	C	C	C	CCC
OC43/human/USA/007-11/2000	C	C	B	CCB
OC43/human/USA/008-5/2000	C	E	E	CEE
**Hong-Kong**				
HK04_01	C	C	C	CCC
HK04_02	B	C	C	BCC *
**Belgium**				
BE03	B	B	B	BBB
BEO4	B	C	C	BCC *
VR759				
HCoV-OC43 (AY391777)	A	A	A	AAA
HCoV-OC43 VR759 (NC005147)	A	A	A	AAA
HCoV-OC43/FRA_EPI/Caen/1967/VR759	A	A	A	AAA

## 3. Discussion

The 4 HCoVs—OC43, 229E, NL63, and HKU1—belong to the viral molecular panel tested during the virological routine diagnostics of acute respiratory infections in humans. Among these four circulating HCoVs, OC43 and NL63 seem to show the greatest prevalence and incidence. These viruses also proved to be the viruses encountered at the earliest age of childhood [43]. These HCoVs circulate widely in epidemic form in the general population, causing infections that are most often benign. Infants, immunosuppressed patients, and very elderly patients may however develop very severe pathologies. These four circulating HCoVs must be distinguished from the emerging HCoVs, SARS-CoV, and MERS-CoV, causes of much graver and potentially fatal respiratory pathologies. Control of the latter HCoVs requires the implementation of international health measures [44,45,46].

The evolutionary potential of coronaviruses brings into play point mutations and recombination events. Such genetic diversity generated in this way may have an impact on the performance of molecular detection techniques used in the virological diagnostic process. The study of intra-specific diversity is thus useful in the monitoring of means of detection. Specifically, it allows for the detection of new circulating variants, and provides information about the evolutionary dynamics of the family of respiratory viruses being monitored, with special attention given to coronaviruses. Our study focuses solely on the HCoV-OC43.

The first analyses of the HCoV-OC43 S gene were conducted in 2005 and revealed a potential spatial and temporal distribution of genetic clusters [34]. In 2011, S. Lau and his colleagues were the first to propose a genotypic classification of HCoV-OC43 from the complete sequencing of three genes: Nsp12 (RdRp), S (spike), and N (nucleocapsid). Four genotypes—A, B, C, and D—were identified: genotype A corresponds to the sequences of the HCoV-OC43 prototype strain VR759, adapted to culture cell line HRT-18 (human adenocarsinoma rectal) or RD (rhabdomyosarcoma); genotypes B and C correspond to contemporary circulating strains, and genotype D is described as a recombinant B/C virus. This study was performed on 29 HCoV-OC43s detected in respiratory samples collected in Hong Kong over a period of 7 years (2004 to 2011), in a non-homogenous temporal distribution. More precisely, the majority of these samples (18 of 29) were collected in 2004, and the others (11 of 29) fall between 2005 and 2011 [33]. Our study defines the genotype of 15 HCoV-OC43s detected from upper respiratory samples frozen at −80 °C, collected over a period of 13 years from 2001 to 2013 at the rate of one to two samples per year. The 15 HCoV-OC43s were detected in patients hospitalized in our university hospital. Of these 15 patients, 10 were male and five were female, at ages from 4 months to 67 years. The symptomatology proved variable (Table 3). The infections were essentially located in upper and lower respiratory airways. The presence of associated gastrointestinal symptoms in some patients should be noted. The prototype strain HCoV-OC43 ATCC VR759, grown on cell line HRT-18, was used as a control, allowing for the validation of the methodology used by Lau *et al.* [33]. This methodology had to be adapted since some of the primers published by the authors did not allow for amplification of all the HCoV-OC43s.

**Table 3:** Clinical features of the 15 french patients. ^a^ Mo., month; yr., year; d., days. ^b^ M, male; F, female. ^c^ Na, not available.

Specimen	Age at Time of Sampling ^a^	Date If Sampling (Day/Mo/Year)	Gender ^b^	Duration of Hospitalization ^c^	Final Diagnosis
Caen/2001/01	3 mo.	02/20/2001	M	na	na
Caen/2001/02	3 yr.	02/07/2011	M	4 d.	gastroenteritis
Caen/2002/03	5 mo.	03/12/2002	M	15 d.	nasoparyngitis, seromucous bilateral otitis
Caen/2002/04	4 mo.	02/21/2002	M	na	na
Caen/2003/05	1 yr.	03/17/2003	M	na	na
Caen/2004/06	1 yr.	02/20/2004	F	4 d.	gastroenteritis
Caen/2005/07	2 yr.	02/07/2005	F	4 d.	gastroenteritis, acute otitis media
Caen/2006/08	11 mo.	04/12/2006	M	na	na
Caen/2007/09	6 mo.	06/16/2007	M	na	na
Caen/2008/10	3 yr.	01/17/2008	M	5 d.	acute etmoidis
Caen/2009/11	3 mo.	11/02/2009	M	3 d.	bronchitis
Caen/2010/12	1 yr.	12/17/2010	M	na	bronchitis
Caen/2011/13	4 mo.	11/20/2010	M	na	na
Caen/2012/14	67 yr.	03/30/2012	M	na	confusion
Caen/2013/15	53 yr.	03/18/2013	F	na	Upper respiratory infection

The alignments of complete sequences of three genes—nsp12, S, and N—of the 15 HCoV-OC43s used in this study were generated and enriched by the addition of the corresponding sequences of several complete HCoV-OC43 genomes available in GenBank: two prototype sequences of HCoV-OC43s VR759 published by Vijgen *et al.* in 2005 [28]; two HCoV-OC43s from 2004 reported by Lau *et al.* in 2011 (HK04_01, HK04_02) [33]; and two HCoV-OC43s described in Belgium in 2003 and 2004 (Belgium 03 and Belgium 04 respectively) [34]. This data set was completed at the end of 2013 with the publication of 41 complete HCoV-OC43 sequences by Town *et al.* in GenBank. These sequences correspond to 41 HCoV-OC43s identified in the USA (Maryland) between 1983 and 2000. Of these 41 sequences, we selected 14 that reflect the genetic diversity and temporal distribution of the whole, and we introduced them into the alignment. In the end, three respective alignments of complete genes—nsp12, S, and N—of 36 HCoV-OC43s identified in three different regions of the world (Hong Kong, Europe, and the USA) over a period of 30 years (from 1983 to 2013) were generated and analyzed. They served as the basis for the construction of phylogenetic trees. Within the three phylogenetic trees—nsp12, S, and N—we identified six different clusters named A, B, C, E, F, and G. A comparative study of the topology of phylogenetic trees corresponding to several genes of the same virus is often used in phylogeny to define recombinant viruses [33,47,48]. We thus compared the topology of nsp12, S, and N trees by observing the contextual position of the corresponding sequences of the same HCoV-OC43 molecular isolate. The complexity of the results of our analysis led us to establish a nomenclature allowing for the simplest expression of the genotypes: each genotype is expressed in the form of a three-letter code, linking each one to the member cluster of the different nsp12, S, and N sequences. From here, we defined 13 different genotypes, of which four were non-recombinant (AAA, BBB, CCC, and EEE). The nine remaining genotypes correspond to different recombinant genotypes (Table 2). Among the 36 HCoV-OC43 sequences examined in this study, 14 HCoV-OC43s of so-called non-recombinant genotypes were found: three HCoV-OC43s of genotype AAA corresponding to prototype strains VR759; three HCoV-OC43s of genotype BBB (origin, France 2003 and 2006, and Belgium 2003); two HCoV-OC43s of genotype CCC (origin, USA 1997 and Hong Kong 2004); and lastly, six HCoV-OC43s of genotype EEE, all originating in the USA, and having circulated from 1983 to 1995. The foremost important point to underline is that these results suggest a high level of recombination among circulating HCoV-OC43 epidemic strains. The limited number of sequences studied in time and space does not allow for conclusive evidence of a possible temporal and spatial distribution of different genotypes HCoV-OC43. However, it should be noted that the E cluster has not yet been described. In the present study, it is identified only in HCoV-OC43 sequences from the USA and France and data suggest that it has been circulating since the 1940s after being the first known group to differentiate from BCoV.

**Figure 3:**
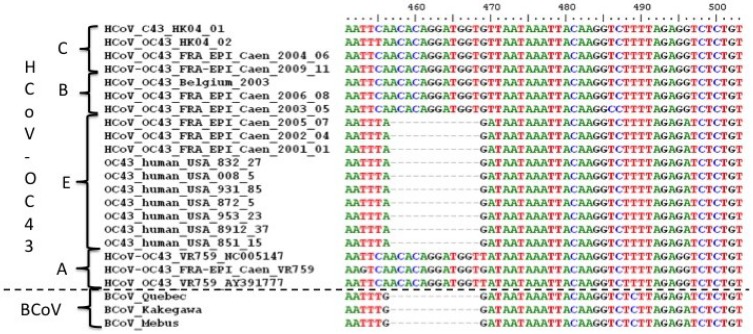
Alignement of some of the S genes sequences. This aligment was made using Bioedit Software version 7.2.5 (Ibis Biosciences, Carlsbad, CA, USA).

The analysis of alignments shows that cluster E is characterized by a deletion of 12 nucleotides in the S gene, more precisely in the S1 gene that codes the glomerular part of the S protein (position 24,091–24,102, GenBank accession number NC_005147). This deletion of 12 nucleotides in the coding phase results in a deletion of 4 amino acids, TQDG, in the corresponding protein sequence. This deletion was also present in the bovine coronavirus strains used in the alignment, as shown in Figure 3. The analysis of 105 S1 BCoV sequences available in GenBank shows that this deletion is expressed in all BCoVs, and described in bovine coronaviruses associated with both enteric and respiratory tropisms. This deletion is therefore not a specific marker of viral tropism. It is located in the lectin domain of the S1 protein of BCoV, and is involved in the attachment of the virus to the cellular receptor, identified as a derivative of neuraminic acid (N-acetyl-9-O-acetylneuraminic acid or Neu5,9Ac2). No protein receptor has yet been described for the BCoV or, more broadly, for *Betacoronavirus* bovine-like clade A. We analyzed all the sequences of this S1 HCoV-OC43 region available in GenBank, for a total of 108 sequences, and the deletion of 12 nucleotides is found in only 27 of them. Of these 27 sequences, 19 correspond to HCoV-OC43s detected in respiratory samples from the USA and sequenced by Town *et al.* Four correspond to HCoV-OC43s detected in France (three in the current study, and one published by our team in 2006); two correspond to HCoV-OC43 sequences deposited by Browlin *et al.* under patent in the United Kingdom (EP2182066 and WO2004011651), and finally, the remaining two correspond to HCoV-OC43s adapted to Vero cells and MDCK I, published in 1996 by F. Künkel and Herrler G [49]. The HCoV-OC43 E cluster is phylogenetically close to the BCoV cluster, and the presence of the genetic marker common to these two clusters provides an argument supporting the hypothesis put forth by Vijgen in 2006 that would identify a crossing of the species barrier occurring from cattle to human [39]. A molecular clock analysis was inferred using the BEAST software version 1.8.1 (http://beast.bio.ed.ac.uk) based on sequence alignments of S and N genes. Sequences of the nsp12 gene were not used because they were uninformative due to the low variability of this gene. Results were compared with those previously obtained by Vijgen from 20 *Betacoronavirus* clade A sequences (nine BCoVs, three PHEVs, and eight HCoV-OC43s). It should be noted that eight sets of HCoV-OC43 sequences used in the analysis of Vijgen *et al.* in 2006 were obtained from samples collected over a very short period between 2003 and 2004 [39].

In 2013, Wertheim *et al.* demonstrated that a strong purifying selection could lead to the underestimation of the evolutionary rate of coronaviruses. In this study, the authors proposed an alternative theory of origin for all coronaviruses. They estimate the tMRCA of coronaviruses infecting mammalian species (*Alpha-* and *Betacoronavirus* genus) and avian species (*Gamma*- and *Deltacoronavirus* genus) at about 293 million years, as opposed to 10,000 years as previously estimated [5]. These authors found a general agreement in the inferred branch lengths between evolutionary models for the shorter branches (inferior to 0.05 substitution per site). As expected in the phylogeny of two species of the same coronavirus genus involved in a relatively recent zoonotic transmission event, all the branches of neighbor joining and Bayesian trees were shorter than 0.03 substitutions per site, as shown in Figure 1 and Figure 2. It can be speculated that the phylogenetic analyses presented in this study did not underestimate the date of emergence of the different clusters nor the estimation of tMRCA for HCoV-OC43 and BCoV. The molecular clock analysis indicated that HCoV-OC43 and BCoV strains have emerged from their tMRCA around 1890 (1859–1952), which was consistent with the data published by Vijgen *et al.* in 2006 [39]. The emergence of cluster E, characterized by a TQGD amino acid deletion, is estimated at around 1944 (1942–1968), and would have occurred at the same time as that of cluster A, in the 1942–1958 time range. The cluster A includes the first HCoV-OC43 strain identified in 1967 from a respiratory sample [13]. The B and C clusters would have circulated later, since the 1980s. The molecular clock results therefore suggest that, since their emergence in the late nineteenth century, the evolution of circulating HCoV-OC43 epidemic strains would have been marked by the occurrence of an insertion of four amino acids, TQDG, in the N-terminal region of the S1 attachment protein, as opposed to the occurrence of the deletion of these four amino acids. The pursuit of this study should lead to a means of determining the functional impact of this insertion, especially with regard to tropism and the clinical expression of infection by HCoV-OC43. Note that the frequent presence of gastrointestinal symptoms in the clinical landscape of HCoV-OC43 respiratory infections has yet to be accounted for.

In 2014, Hu *et al.* studied the genetic diversity of the HCoV-OC43 circulating in Beijing, China [50]. The authors performed partial sequencing of the S1 and N genes (24,228–24,785 and 30,022–30,580 for S1 and N respectively, using the HCoV-OC43 sequence associated with the accession number NC_005147 as reference) of 50 HCoV-OC43s detected in the respiratory samples, all taken in 2012. They included the sequences published by Lau *et al.* in their analysis in 2011, and used the nomenclature established by these same authors to identify various clusters obtained [33,50]. In addition to the A and B clusters, they identified 4 other groups, designated UNT1, UNT(B), UNT(C/D), and (C/D), respectively. Several clusters were found to correspond to those previously described by Lau *et al.* in 2011 [33]. The UNT(B) cluster or “Untyped B” is a group close to the B cluster. The C/D cluster corresponds to the C cluster, named “C/D”, due to the presence of the HK04_02 strain defined by Lau *et al.* as a genotype D, referring to a recombination virus belonging to the B cluster on the nsp12 sequence and to the C cluster on the S and N sequences [33]. The UNT(C/D) cluster or “Untyped (C/D)” corresponds to a group close to the preceding cluster. Finally, the UNT1 cluster or “Untyped 1”, which is close to the BCoV sequences included in this analysis, might correspond to the E cluster describe in the current study. In the report by Hu *et al.* (2014), the UNT1 cluster includes only five HCoV-OC43s (accession numbers Z32769, HC734573, CQ772300, DD266155, and Z32768) [50]. We have verified that they all have the characteristic nucleotide deletion of 12 nucleotides. This very recent report supports the results determined in the current study in that they both demonstrate a high genetic diversity of circulating HCoV-OC43s.

The present study highlights the difficulty of investigating intraspecific genetic variability of HCoV-OC43s, though the analysis of partial viral genomes (complete genes) allows the description of clear recombination patterns. Nevertheless, due to the high evolutionary potential of HCoV-OC43, analyzing complete genomes would reveal a more detailed and perhaps more complex recombination pattern. In addition, the data reported and analyzed here illustrate the dynamic diversification of HCoV-OC43 following its emergence in human. This remarkable diversification contrasts with the relative stability of BCoV in cattle (unpublished data). The present study therefore draws attention to the importance of increasing our knowledge of the evolution dynamism of coronavirus in combating the permanent emergence risk that they pose in the human population, evidenced by the SARS pandemic of 2002–2003 and the recently identified MERS-coronavirus in the Middle-East.

## 4. Materials and Methods

### 4.1. Clinical Samples and Positive Control

Fifteen human respiratory specimens (upper and lower) positive for HCoV-OC43 using molecular detection, sampled from 2001 to 2013 (one to two for each year) were chosen among the specimens available at the virology department of the University Hospital of Caen. These specimens were sampled from patients suffering from acute upper or lower respiratory diseases, and were sent to our laboratory for a viral diagnosis. The detection of HCoV-OC43 was performed using an in-house real-time quantitative RT-PCR, for which the original primers may be found in Table 4. Clinical signs, age, and sex of patients were investigated and compiled in Table 3. We also used a culture supernatant of the prototype strains VR759 as reference and for validation of our method. This strain was obtained from the ATCC and maintained in HRT-18 cells.

**Table 4:** Primer and probes used for RT-PCR and full sequencing of nsp12, S, and N genes of 15 HCoV-OC43s, and the prototype strain VR759.

Real-Time RT-PCR					
Primer or Probe Name	Polarity	Primer/Probe Sequences (5'-3')	Gene Target	Location (5'-3')	Reference
LOC1	fwd	GTGGTTTTGCTGTTTATGTTAAGT	N	28991-29014	this study
LOC2	rev	AGATATTATTTCTCAACAATGCGGT	N	29092-29068	this study
SLOC	fwd	HEX-AATTACCGACTGCCATCAACCCAAA-BHQ1	N	29026-29050	this study
**RT-PCR and sequencing**					
**Primer name**	**Polarity**	**Primer sequences (5'-3')**	**Gene target**	**Location (5'-3')**	**Reference**
OC43_P_13158F	fwd	TTGTGCAAATTACGCGGCAA	RdRp	13171-13190	this study
OC43_P_13896R	rev	TTCACCAATTTGTCCGCAAAC	RdRp	13907-13889	this study
OC43_P_13645F	fwd	GCCACACATTGTACGCAAGG	RdRp	13649-13668	this study
OC43_P_14511R	rev	CCGCTTGTTATAGCGGCAAC	RdRp	14515-14496	this study
OC43_P1_4375F	fwd	GTGGATACACATCGTTATCGCTT	RdRp	14379-14401	this study
OC43_P_15253R	rev	CCTATCGCTTTGCGAACAACA	RdRp	15257-15237	this study
LPW 3064F	fwd	CTGGGATGATATGTTACGCCG	RdRp	15095-15115	Lau *et al.* 2011
LPW 2579R	rev	GTGTGTTGTGAACARAAYTCRTG	RdRp	15763-15750	Lau *et al.* 2011
OC43_P_15276F	fwd	TGAGTGATGATGGGGTTGTGT	RdRp	15577-15597	this study
OC43_P_16129R	rev	TTGAGAAGAGCAGACCACGC	RdRp	16133-16114	this study
OC43_P_15814F	fwd	AGGAGCTGGATGTTTTGTAGATGA	RdRp	15818-15841	this study
LPW 1127R	rev	TGCCTTTTGCGTTTCTGC	RdRp	16520-16503	Lau *et al.* 2011
LPW 1162F	fwd	CCYRTTTGTRTGTATGATCC	S	23500-23520	Lau *et al.* 2011
LPW 1166R	rev	YGCATAAAAAGTACCACC	S	24325-24307	Lau *et al.* 2011
OC43_S_23506F	fwd	TGTGTGTATGATCCGCTACCAG	S	23507-23528	this study
OC43_S_24384R	rev	GTGAAAGYGCCATGCCTAAA	S	24391-24372	this study
LPW 6447F	fwd	CTTCAAAGAACTATGGCATT	S	24198-24217	Lau *et al.* 2011
LPW 6548R	rev	GACTGCAAATAGCCCAAATT	S	24917-24898	Lau *et al.* 2011
LPW 2095F	fwd	TGATGCTGCTAAGATATATGG	S	24804-24824	Lau *et al.* 2011
OC43_S_24569R	rev	AACCTCAACAAAAATGCCTTGG	S	25581-25560	this study
OC43_S_25372F	fwd	AGCATTTTTGGGTTGGTCTGC	S	25383-25402	this study
OC43_S_26126R	rev	AATCACCACAGACAAATGCAGC	S	26137-26116	this study
OC43_S_25882F	fwd	AGGTAGTGGTTACTGTGTGGA	S	25893-25913	this study
LPW 1178R	rev	GACACCAAGMCCATTAAT	S	26652_26635	Lau *et al.* 2011
OC43_S_26406F	fwd	TGATGTCGGTTTTTGTTGAGGC	S	26418-26438	this study
OC43_S_27152R	rev	CAGACCGGGACTAACCTTCG	S	27162-27143	this study
OC43_S_26971F	fwd	TGCAGCACAAGCTATGGAGA	S	26982-27001	this study
LPW 1275F	fwd	TRAAATGGCCTTGGTATGT	S	27548-27566	Lau *et al.* 2011
LPW 1189R	rev	TKWMWAGGAACTCTACAATA	S	27942-27923	Lau *et al.* 2011
OC43_N_28778F	fwd	TGTTAGGCCGATAATTGAGGACT	N	28803-28825	this study
OC43_N_28728F	fwd	AACCCAGAAACAAACACY	N	28753-28769	this study
OC43_N_29546R	rev	GCGGTCCTGTTCCCAGATAG	N	29500-29481	this study
OC43_N_29232F	fwd	CAGCAACCATCAGGAGGGAA	N	29257-29276	this study
OC43_N_30104R	rev	AAACATCCTTCTGGGGCTG	N	30148-30130	this study
OC43_N_29158F	fwd	CCGATCAGTCCGACCAGTTT	N	29183-29202	this study
OC43_N_30084R	rev	TCTGCACTTTGGCCAACTCT	N	30109-30090	this study
OC43_Nd_29879F	fwd	GAATAARCCCCGCCAGA	N	29904-29920	this study
OC43_N_30656R	rev	CATGCTGGCTCTTTCCCTTG	N	30675-30655	this study
LPW 1195F	fwd	GAGAGGCCCTAATCAGAA	N	29967-29984	Lau *et al.* 2011
OC43_N_30690R	rev	TTAACTTCATTCATTTACTA	N	30715-30696	this study

### 4.2. RNA Extraction and Quantitative RT-PCR

Total nucleic acids of 300 μL of clinical specimens and VR759 cultures supernatant were extracted using the QiAsymphony automate and the QIAsynphony virus/bacteria kit (Qiagen, Holden, Germany), following instructions of manufacturer.

### 4.3. Sequencing of the 3 Complete Genes Nsp12, S and N

To determine the genomic sequence of nsp12, S, and N genes, a set of 17 overlapping RT-PCR products, from 600 to 900 nucleotides, were generated. These RT-PCR products encompassed the entire three genes. Twelve primers were used to amplify and sequence the nsp12 gene, composed of 2783 nucleotides and located from nucleotides 13,317 to 16,099 (reference to VR759, NC005147). Eighteen primers were used to amplify and sequence the S gene composed of 4062 nucleotides. This gene is located in the last third of the HCoV-OC43 genome, between bases 23,644 and 27,729 (reference to HCoV-OC43 VR759, NC005147). Six primers were used to amplify and sequence the N gene composed of 1347 nucleotides and located at the 3’ end of the HCoV-OC43 genome, from nucleotide 29,104 to 30,450 (reference to HCoV-OC43 VR759, NC005147). For both RT-PCR and sequencing reactions, primers were selected in conserved domains from an alignment of the previously published HCoV-OC43. Some of the selected primers were published by Lau in 2011, but others were designed using Primer-Blast software (http://www.ncbi.nlm.nih.gov/tools/primer-blast/) [33]. All of them were tested *in silico* and *in vitro* with the VR759 prototype strain to test their efficacy. *In vitro* experimentations have also been conducted using the VR759 strain to test the primer abilities to provide us with quality sequencing products. Alternative original primers were used for HCoV-OC43/FRA_EPI/Caen/2001/02, HCoV-OC43/FRA_EPI/Caen/2002/04, HCoV-OC43/FRA_EPI/Caen/2007/09, HCoV-OC43/FRA_EPI/Caen/2001/01, and HCoV-OC43/FRA_EPI/Caen/2002/03. The sequences of the 42 primers used for RT-PCR and sequencing are given in Table 4, associated with the corresponding targeted gene and with the fragment size. RT-PCR reactions were performed using OneStep RT-PCR System (Qiagen, Holden, Germany), from 2.5 μL of nucleic acid in a final volume of 25 μL. The same primers were used to perform the complete bidirectional sequencing of the RT-PCR products, with a Sanger derived method. 2.5 μL of each RT-PCR product were first purified by adding 1 μL of ExoSAP-IT (USB corporation, Cleveland, OH, USA), and by heating it at 37 °C for 15 min, followed by 15 min at 80 °C to disable enzymatic activities. Two sequencing reactions were performed for each RT-PCR product with either a forward or a reverse primer. 1 to 2 μL of purified RT-PCR product were used in a final volume of 10 μL of reaction mixture, made up with the BigDye terminator cycle sequencing kit, version 1.1 (Applied Biosystems, Foster City, CA, USA), in addition to 1 μL of one of the corresponding primers. The cycling parameters for the sequencing RT-PCR were 96 °C for 1 min, followed by 30 cycles of 96 °C for 10 s, 60 °C for 5 s, and 60 °C for 4 min. The analysis of labelled products was performed by the capillary electrophoresis ABI Prism 3500 genetic analyzer (Applied Biosystems, Foster City, CA, USA), by the molecular genetics laboratory of the University Hospital of Caen. In this study, the term of molecular isolate was use to designate the sequence of HCoV-OC43 detected directly from clinical sample.

### 4.4. Bioinformatic Analysis

Sequences were assembled in contigs corresponding to the entire nsp12, S or N genes with CodonCode Aligner Software, version 5.0.1 (CodonCode corporation, Centerville, MA, USA). Multiple sequence alignment and phylogenetic analysis were performed using MEGA software, version 6.06 (http://www.megasoftware.net). Twenty-one sequences available in GenBank were added to the alignment, including HCoV-OC43s HKU4_01 and HKU4_02 from Hong-Kong [33]; HCoV-OC43s BE03 and BE04 from Belgium [34]; HCoV-OC43s OC43/human/USA/851-15/1985, OC43/human/USA/007-11/2000, OC43/human/USA/925-1/1992, OC43/human/USA/8912-37/1989, OC43/human/USA/953-23/1995, OC43/human/USA/951-18/1995, OC43/human/USA/872-5/1987, OC43/human/USA/901-54/1990, OC43/human/USA/931-85/1993, OC43/human/USA/911-58/1991, OC43/human/USA/008-5/2000, OC43/human/USA/832-27/1983, OC43/human/USA/965-6/1996 and OC43/human/USA/971-5/1997 from the USA, recently submitted by Town *et al.*; and BCoV Kakewaga, Mebus, and Quebec to root the trees [36,38,51]. The accession numbers corresponding to these sequences are given in Table 3. Phylogenetic trees were constructed using the method of neighbor joining, with the substitution model of Kimura-2, implemented in MEGA6 [41,52]. These trees were used to perform the comparative analysis of our results with those of Lau *et* al in 2011 [33]. In order to support neighbour joining trees with a probabilistic method and to estimate divergence time between groups and subgroups, trees of S and N genes were also inferred using the same sequences and a Bayesian Markov Chain Monte Carlo method, implemented in the BEAST package, version 1.8.1 [42,53]. Inferences were calculated with the one parametric coalescent model with a constant size, under the TN93+G substitution model according to Bayesian Information Criterion (BIC) and Akaike Information Criterion (AIC) values of a model test carried out on MEGA6 software. The tMRCA was estimated using a relaxed molecular clock with an uncorrelated lognormal distribution. The length of MCMC was fixed at 3 × 10^−8^ states for the both genes. The Tracer software, version 1.5, implemented in the BEAST package, was used to assess the fitness of the model used, focusing the Effective Sampling Size (ESS) data, after a burning of 10%. The target trees were obtained with the maximum credibility clades, with a posterior probability limit of 0.05. The tMRCA age for each genotype was compared for S and N genes.
